# Trends and socioeconomic-spatial inequalities in hypertension among Muslim women in India, 2015–2021: evidence from the National Family Health Surveys

**DOI:** 10.3389/fpubh.2026.1828079

**Published:** 2026-06-11

**Authors:** Zeenat Hashmi, Ashish Singh

**Affiliations:** Shailesh J. Mehta School of Management, Indian Institute of Technology Bombay, Mumbai, India

**Keywords:** epidemiological transition, hypertension, Muslim women, social determinants of health, socioeconomic inequality, spatial epidemiology, women’s health

## Abstract

**Introduction:**

Hypertension is a growing public health concern in India, but little is known about how socioeconomic and spatial inequalities shape hypertension risk within religious minority populations. This study examines trends and inequalities in hypertension among Muslim women aged 15–49 years in India.

**Methods:**

The analysis uses nationally representative data from NFHS-4 (2015–16) and NFHS-5 (2019–21). Hypertension was defined as systolic blood pressure ≥140 mmHg and/or diastolic blood pressure ≥90 mmHg, or current use of antihypertensive medication. Weighted prevalence estimates, Poor-Rich ratios, Concentration Indices, survey-weighted logistic regression models, Moran’s I, and LISA statistics were used to assess socioeconomic and spatial inequalities.

**Results:**

Hypertension prevalence among Muslim women increased from 12.11% in NFHS-4 to 12.52% in NFHS-5. A strong age gradient persisted: compared with women aged 15–19 years, women aged 40–49 years had substantially higher adjusted odds of hypertension in both NFHS-4 (OR = 9.19) and NFHS-5 (OR = 8.33). In NFHS-5, higher education was associated with lower odds of hypertension (OR = 0.65; 95% CI: 0.50–0.84), while women in the fourth and richest wealth quintiles had higher odds than the poorest quintile (OR = 1.51; 95% CI: 1.22–1.86 and OR = 1.37; 95% CI: 1.07–1.74, respectively). Concentration Index values remained positive for several subgroups, including secondary-educated women (0.104 to 0.125) and employed women (0.042 to 0.123), indicating continued concentration among relatively better-off groups. District-level spatial clustering was statistically significant among Muslim women, with Moran’s I increasing from 0.111 in NFHS-4 to 0.210 in NFHS-5 (*p* < 0.001).

**Discussion:**

Hypertension among Muslim women is socially and spatially patterned, with evidence of age-related risk, shifting wealth gradients, and persistent geographic clustering. The findings highlight the need for hypertension screening and prevention strategies that account for socioeconomic position, minority status, and subnational spatial inequality.

## 1 Introduction

Hypertension has become one of the most important non-communicable disease risks in low- and middle-income countries, including India. Although often asymptomatic, it is strongly associated with cardiovascular disease, stroke, kidney disease, and premature mortality. Recent global estimates indicate that more than 1.4 billion adults live with hypertension, with a large share concentrated in low- and middle-income countries where detection, treatment, and control remain limited (1). In India, the rising burden of hypertension reflects a wider epidemiological transition in which chronic diseases increasingly coexist with persistent undernutrition, infectious disease burdens, and socioeconomic inequality (2–5, 41, 44).

A growing literature has examined hypertension in India, but most studies focus on the general adult population or include religion only as a control variable (6). This limits understanding of how chronic disease risks are distributed within religious minority populations. Muslim women are an important group in this regard. Muslims constitute India’s largest religious minority and have experienced persistent disadvantages in education, employment, housing, and access to public services (7, 8). For women, these disadvantages may intersect with gendered constraints on mobility, labor force participation, household decision-making, and access to healthcare (9, 10). Hypertension among Muslim women of reproductive age is therefore not only a clinical concern, but also an indicator of how chronic disease risk is socially patterned during India’s ongoing health transition.

Existing research also gives limited attention to how socioeconomic gradients in hypertension may change over time. During the early stages of chronic disease transition, hypertension may be more visible among relatively better-off groups because of lifestyle change, sedentary work, urban residence, psychosocial stress, and better access to screening. As risks diffuse across the population, these gradients may flatten, become non-linear, or partially reverse (11, 12). However, whether such gradient dynamics are visible among Muslim women in India remains insufficiently examined. Similarly, spatial inequalities in hypertension have received increasing attention, but few studies combine socioeconomic inequality measures with district-level spatial clustering for a religious minority population.

This paper addresses these gaps using data from the fourth and fifth rounds of the National Family Health Survey, conducted in 2015–16 and 2019–21. The analysis examines trends and socioeconomic-spatial inequalities in hypertension among Muslim women aged 15–49 years in India. It uses weighted prevalence estimates, Poor-Rich ratios, Concentration Indices, survey-weighted logistic regression models, Moran’s I, and Local Indicators of Spatial Association. The study is designed as a within-group analysis of Muslim women rather than as a formal comparison across religious communities. Accordingly, the paper examines how hypertension is patterned by age, education, wealth, employment, marital status, residence, and region within Muslim women, while interpreting broader minority-status-linked pathways cautiously.

The paper makes three main contributions. First, it provides nationally representative evidence on hypertension among Muslim women in India, a group that remains underrepresented in non-communicable disease research. Second, it examines whether wealth and education gradients in hypertension show signs of flattening, non-linearity, or partial reversal between NFHS-4 and NFHS-5. Third, it integrates socioeconomic inequality measures with spatial analysis to show how hypertension risk is distributed across both social and geographic dimensions. By doing so, the study offers a more disaggregated account of chronic disease inequality during India’s epidemiological transition.

## 2 Conceptual framework and literature review

### 2.1 Health gradients and epidemiological transition

A central insight of population economics is that health outcomes are systematically patterned by socioeconomic status. Across countries and within societies, individuals with higher income, wealth, and education tend to experience better health and longer life expectancy, a relationship commonly described as the health gradient (2, 5, 12). This gradient reflects multiple channels, including differences in material resources, knowledge, health behaviors, working conditions, and access to healthcare. Importantly, the health gradient is not a static feature of societies; rather, it evolves with economic development and demographic change.

Epidemiological transition theory provides a complementary framework for understanding these dynamics (43). In its classic formulation, the transition describes a shift from high mortality due to infectious diseases and nutritional deficiencies toward a disease burden dominated by chronic and degenerative conditions as societies develop (4). Subsequent economic interpretations emphasize that this transition is uneven and socially differentiated, shaped by changes in income, urbanization, labor markets, and institutional capacity (2, 5). Evidence from India shows persistent socioeconomic inequalities in health and longevity across caste, religion, wealth, and region, even during periods of economic growth (13). Chronic conditions such as hypertension are emblematic of this transition, emerging as populations live longer and are increasingly exposed to sedentary lifestyles, dietary change, and psychosocial stress.

During periods of rapid transition, the relationship between socioeconomic status and health may weaken or change direction. A growing literature documents instances of health gradient flattening or reversal, whereby higher socioeconomic groups experience equal or greater prevalence of certain chronic conditions compared to poorer groups (11, 12, 14). In early stages of transition, lifestyle-related risks often appear first among wealthier and more educated populations, who are more likely to work in sedentary occupations, consume energy-dense diets, and live in urban environments. At the same time, better access to healthcare and screening can increase detection among advantaged groups, further amplifying observed prevalence. Over time, as these risks diffuse more broadly, gradients may flatten or reverse again, underscoring the dynamic nature of health inequality.

These theoretical insights motivate the use of distribution-sensitive measures to assess health inequality. Poor-Rich ratios and concentration indices capture not only differences in average prevalence but also the degree to which health outcomes are concentrated among advantaged or disadvantaged groups (15, 16). Applied to hypertension, such measures allow an assessment of whether cardiovascular risk is increasingly borne by higher socioeconomic strata, consistent with early-stage epidemiological transition, or whether it remains concentrated among the poor. This framework underpins the empirical strategy adopted in this study.

### 2.2 Socioeconomic patterning of hypertension among Muslim women

While epidemiological transition theory and health-gradient models provide a broad account of how development reshapes disease patterns, they often pay less attention to the social locations through which economic change is experienced. Religious minority status is one such location. In this paper, religion is not treated as a direct biological or cultural determinant of hypertension. Rather, the focus is on how Muslim women’s social and economic location in India may shape exposure to socioeconomic, gendered, and spatial conditions associated with cardiometabolic risk.

In India, Muslims constitute the largest religious minority and have historically experienced disadvantages in education, employment, housing, and access to public services relative to the majority population (7, 8). For women, these disadvantages may intersect with gendered constraints on mobility, labor force participation, household decision-making, and access to healthcare (9, 10). These conditions do not imply that religion itself causes hypertension. Instead, they suggest that the socioeconomic patterning of hypertension among Muslim women may be shaped by unequal exposure to education, wealth, employment, marital status, residential context, and health-system access (45).

**Figure 1:**
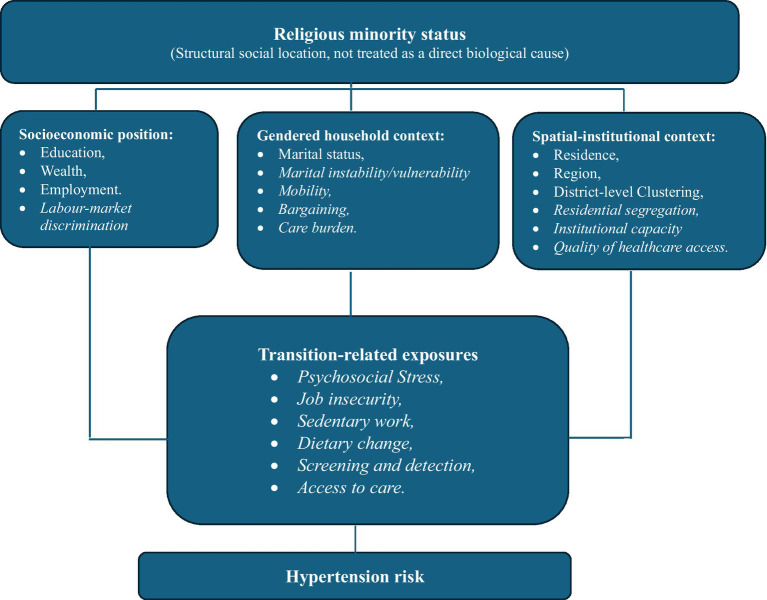
Conceptual pathway linking religious minority status, socioeconomic position, spatial context, and hypertension risk among Muslim women in India. Note: Solid text indicates variables observed in NFHS and included in the empirical analysis; *italic* text indicates theoretically plausible pathways that are not directly tested as mediators in the present study.

Figure 1 presents the conceptual pathway guiding the analysis. We conceptualize religious minority status not as a direct biological or cultural determinant of hypertension, but as a structural social location that may shape exposure to socioeconomic, gendered, and spatial conditions associated with cardiometabolic risk. In this framework, minority status may operate through unequal educational and labor-market opportunities, labor-market discrimination, residential concentration or segregation, gendered restrictions on mobility and household bargaining, marital instability or vulnerability, and differential access to healthcare and screening. However, the NFHS data do not permit direct testing of all these mechanisms. The empirical analysis directly examines observable socioeconomic and demographic correlates, including education, wealth, employment status, marital status, residence, caste, and region, as well as district-level spatial clustering. Mechanisms such as labor-market discrimination, residential segregation, job insecurity, psychosocial stress, intra-household bargaining, care burden, sedentary work, dietary change, and healthcare access or quality are therefore treated as theoretically plausible pathways rather than directly estimated mediators.

Economic theories of household behavior further help interpret these pathways. Household allocation models emphasize that women’s health is shaped not only by income, but also by bargaining power, resource distribution, time allocation, marital status, and social support (17–19). In this sense, employment may not always operate as a protective factor if it is accompanied by insecure work, limited autonomy, or a double burden of paid and unpaid work. Similarly, marital disruption may be associated with economic insecurity and psychosocial strain, particularly in contexts where social protection is limited. Since the NFHS does not directly measure job insecurity, discrimination, intra-household bargaining, or psychosocial stress, these mechanisms are interpreted cautiously as plausible explanations rather than empirically tested channels.

Accordingly, the analysis is designed as a within-group study of Muslim women rather than as a formal comparison between religious communities. It does not estimate a counterfactual difference between Muslim and Hindu women, nor does it test whether socioeconomic gradients differ statistically across religious groups. Instead, the study examines how hypertension is patterned by wealth, education, employment, marital status, residence, and region within Muslim women. This within-group focus allows closer attention to heterogeneity among a population that remains underrepresented in research on non-communicable disease inequalities in India, while also requiring caution in making broader claims about religion as an independent modifying factor.

### 2.3 Spatial inequality, institutions, and health diffusion

Economic and epidemiological transitions are also spatially uneven. The diffusion of health risks across regions reflects differences in urbanization, labor markets, infrastructure, and state capacity, as well as variation in access to healthcare and disease surveillance. From the standpoint of population economics and economic geography, spatial clustering of health outcomes is indicative of uneven development trajectories and institutional disparities rather than random variation (2, 20).

In the context of chronic diseases, spatial inequality can arise through multiple channels. Regions that experience earlier or more rapid economic growth may be exposed sooner to transition-related risks such as sedentary employment and dietary change, while also benefiting from better diagnostic capacity. Conversely, areas with weaker healthcare infrastructure may exhibit lower measured prevalence despite substantial underlying risk. For minority populations, spatial patterns may further reflect residential segregation, concentrated disadvantage, and differential access to public services, reinforcing within-group inequality.

Empirical studies on India have documented substantial regional variation in hypertension prevalence and control, often aligned with broader patterns of economic development and governance (21, 22, 42). However, few analyses have examined whether these spatial patterns differ systematically for religious minorities or how spatial clustering interacts with socioeconomic gradients within such groups. Incorporating spatial methods such as Moran’s I and Local Indicators of Spatial Association (LISA) enables the identification of geographic clusters of high or low prevalence, providing insight into the institutional and economic contexts in which health transitions unfold.

By integrating spatial analysis with socioeconomic inequality measures, this study situates individual-level health outcomes within their broader regional and institutional environments. This approach aligns with the view that health inequality during epidemiological transition is shaped by the interaction of economic status, social structure, and place. It also provides a basis for interpreting spatial clustering not as a cultural artifact but as evidence of uneven development and differential institutional capacity affecting Muslim women’s health.

## 3 Data and methodology

### 3.1 Data source and study population

This study uses data from the fourth and fifth rounds of the National Family Health Survey of India, NFHS-4 (2015–16) and NFHS-5 (2019–21), conducted by the International Institute for Population Sciences under the Demographic and Health Survey program. The NFHS provides nationally representative information on demographic characteristics, socioeconomic conditions, health outcomes, and biomarker measurements for women of reproductive age across Indian states and union territories (23, 24).

The women’s individual recode files included 699,686 women in NFHS-4 and 724,115 women in NFHS-5. Among them, 94,591 women in NFHS-4 and 90,729 women in NFHS-5 were Muslim. The primary analytical focus of this study is Muslim women aged 15–49 years with valid information on hypertension status. Observations with missing or invalid blood pressure readings, missing information on current antihypertensive medication use, or missing values for variables used in a given model were excluded from the relevant analysis.

Although selected estimates for all women are presented to provide broader contextual and spatial comparison, the main descriptive, inequality, and regression analyses focus on Muslim women.

All descriptive and regression analyses used the NFHS women’s sampling weights. The weights were divided by 1,000,000 before analysis. The complex survey design was accounted for using the primary sampling unit and stratification variables provided in the NFHS datasets, with clustering at the PSU level and stratification by the NFHS strata variable.

### 3.2 Measurement of hypertension

#### 3.2.1 Blood pressure measurement protocol

In both NFHS-4 and NFHS-5, systolic and diastolic blood pressure were measured using a standardized digital blood pressure monitor administered by trained health investigators. Following the NFHS/DHS biomarker protocol, up to three blood pressure readings were taken for each eligible respondent in a seated position, with a five-minute interval between successive readings. The first reading was not used to construct the main blood pressure measure because first readings may be affected by initial measurement anxiety or a temporary elevation (23, 24).

The analytic measure was therefore constructed using the average of the second and third valid systolic and diastolic readings. Where only two valid readings were available, the average of the two valid readings was used; where only one valid reading was available, that reading was retained. Implausible readings outside biologically valid ranges were excluded prior to analysis. This approach follows the NFHS/DHS measurement protocol and is widely used in population-based hypertension research using NFHS data (25, 26).

#### 3.2.2 Definition and severity of hypertension

Hypertension was defined as systolic blood pressure of 140 mmHg or higher, and/or diastolic blood pressure of 90 mmHg or higher, or current use of antihypertensive medication at the time of the survey. This definition follows widely used clinical and epidemiological thresholds and ensures comparability across NFHS rounds and with other population-based studies (25, 26).

To describe the intensity of elevated blood pressure, measured hypertension was further classified into mild, moderate, and severe categories. Mild hypertension was defined as SBP 140–159 mmHg or DBP 90–99 mmHg; moderate hypertension as SBP 160–179 mmHg or DBP 100–109 mmHg; and severe hypertension as SBP ≥ 180 mmHg or DBP ≥ 110 mmHg. Where systolic and diastolic readings fell into different categories, the respondent was classified according to the higher severity category. These categories are used descriptively to show the distribution and intensity of elevated measured blood pressure across socioeconomic groups.

### 3.3 Socioeconomic and demographic variables

Socioeconomic and demographic characteristics were selected based on population-economic theories of health gradients and household allocation. Age was categorized into four groups (15–19, 20–29, 30–39, and 40–49 years). Educational attainment was grouped as no education, primary, secondary, and higher education. Household economic status was measured using the NFHS wealth index, a composite indicator derived through principal component analysis of household assets and amenities, and categorized into quintiles (27).

Additional covariates included caste (Scheduled Castes/Scheduled Tribes, Other Backward Classes, and others), place of residence (urban/rural), employment status, marital status (never married, currently married, formerly married), and region of residence.

Employment status was coded as a binary variable distinguishing currently employed and not employed women. This measure captures whether a woman was engaged in work, but it does not distinguish between formal salaried employment, casual labor, agricultural work, self-employment, home-based work, or unpaid family work. These forms of work may involve different levels of physical demand, income security, autonomy, time pressure, and psychosocial stress. Therefore, employment status is interpreted as a broad marker of labor force participation rather than as a direct measure of employment quality or occupational conditions.

Marital status was categorized as never married, currently married, and formerly married. The “formerly married” category includes women who were widowed, divorced, or separated at the time of the survey. These categories were combined in the main analysis to maintain statistical stability and comparability across NFHS-4 and NFHS-5, particularly because the overall share of formerly married Muslim women is relatively small. However, widowhood, divorce, and separation may represent different social, economic, and psychosocial circumstances. The “formerly married” category is therefore interpreted as a broad indicator of marital disruption rather than as a homogeneous marital state.

These variables capture multiple dimensions through which religion intersects with economic position, gender roles, and spatial context.

## 4 Methods

### 4.1 Descriptive analysis

Weighted prevalence estimates of hypertension and its severity were computed across socioeconomic and demographic groups for both NFHS rounds. These estimates provide an initial assessment of age gradients, education gradients, wealth differentials, and temporal changes in prevalence.

### 4.2 Poor-rich ratios

Socioeconomic inequality in hypertension was first assessed using Poor-Rich (PR) ratios, defined as the ratio of hypertension prevalence among women in the poorest wealth quintile to that among women in the richest quintile. PR ratios offer transparent measure of relative inequality and are particularly useful when disease prevalence is high, where odds ratios may overstate relative differences (28, 29).

The PR ratio is useful because it provides an intuitive comparison between the two ends of the wealth distribution. However, it does not capture patterns within the middle wealth quintiles. A PR ratio close to or below unity therefore does not necessarily imply the absence of socioeconomic inequality; it only indicates that the prevalence among the poorest group is similar to or lower than that among the richest group. Non-monotonic patterns across intermediate quintiles may remain hidden when relying only on this extreme-group comparison.

PR ratios have been widely applied in population health research in India to track changes in socioeconomic gradients over time and across population subgroups (30, 31).

Values approaching unity indicate gradient flattening, while values below unity suggest emerging reversal, where risk becomes concentrated among economically better-off groups.

### 4.3 Concentration index

To provide a summary measure of income-related inequality across the full socioeconomic distribution, the Concentration Index (CI) was estimated for hypertension prevalence. The CI quantifies the extent to which a health outcome is disproportionately concentrated among individuals ranked by economic status. Unlike the PR ratio, the Concentration Index uses information from the full socioeconomic distribution. It is therefore sensitive not only to differences between the poorest and richest quintiles, but also to how hypertension is distributed across intermediate wealth groups. A positive CI can coexist with a PR ratio close to or below one if prevalence is higher among middle or richer quintiles than among both the poorest and richest groups. This distinction is important for interpreting non-linear or non-monotonic wealth gradients in hypertension. A positive CI indicates concentration among the better- off, while a negative value reflects concentration among the worse-off (32, 46).

Concentration indices were calculated separately by selected socioeconomic characteristics and survey rounds, allowing assessment of whether inequality persisted, widened, or converged during the study period. Standard errors were estimated accounting for the complex survey design using established methods for concentration indices (15).

### 4.4 Multivariable regression analysis

Multivariable logistic regression models were estimated to examine adjusted associations between socioeconomic characteristics and hypertension. Separate models were fitted for NFHS-4 and NFHS-5 to allow temporal comparison. The dependent variable was binary hypertension status, and independent variables included age, education, wealth quintile, employment status, marital status, caste, place of residence, and region.

Adjusted odds ratios are reported with corresponding confidence intervals. These models are intended to identify correlates and structural associations rather than causal effects, consistent with the emphasis on interpretation within broader economic and demographic processes.

Survey-weighted logistic regression models were estimated using NFHS sampling weights, with adjustment for clustering at the primary sampling unit level and stratification using the NFHS strata variable. Observations with missing values for the dependent variable or any covariate included in the model were excluded through listwise deletion. The models are interpreted as adjusted associations rather than causal estimates.

### 4.5 Spatial analysis

#### 4.5.1 Global spatial autocorrelation

District-level hypertension prevalence was mapped to examine geographic variation across India. Global spatial autocorrelation was assessed using Moran’s I, which measures whether prevalence values are spatially clustered, dispersed, or randomly distributed across districts (33). A first-order contiguity spatial weights matrix was used to define neighboring districts.

#### 4.5.2 Local indicators of spatial association (LISA)

To identify localized clusters of high and low hypertension prevalence, Local Indicators of Spatial Association (LISA) were estimated. LISA statistics classify districts into high-high, low- low, high-low, and low-high clusters, enabling identification of spatial hotspots and cold spots (34).

Separate LISA analyses were conducted for all women and Muslim women, and for both NFHS rounds, allowing examination of whether spatial clustering patterns differ by religion and over time. Spatial analysis complements socioeconomic inequality measures by highlighting institutional, infrastructural, and regional dimensions of health inequality. District-level hypertension prevalence estimates were calculated using NFHS sampling weights. For the spatial analysis among Muslim women, districts with unavailable estimates or insufficient observations were excluded from Moran’s I and LISA classification and are shown in grey in the maps. Because the analysis is disaggregated by religion at the district level, some Muslim subsamples may be small, and district-specific prevalence estimates may therefore be less precise than state- or region-level estimates. The spatial analysis is consequently interpreted as identifying broad clustering patterns rather than producing precise district-level prevalence rankings.

## 5 Results

This section presents findings on hypertension prevalence, socioeconomic gradients, inequality measures, adjusted associations, and spatial clustering among women aged 15–49 years in India, with the primary focus on Muslim women. Results are presented sequentially, beginning with descriptive patterns and then moving to inequality measures, regression results, and spatial analysis.

### 5.1 Trends and socioeconomic gradients in hypertension

Table 1 presents the distribution of selected socioeconomic and demographic characteristics of women aged 15–49 years in NFHS-4 and NFHS-5, with particular attention to the Muslim women included in the analysis. The share of Muslim women in the sample remained broadly stable across the two rounds, declining slightly from 13.79% in NFHS-4 to 13.48% in NFHS-5. Within the Muslim women’s sample, educational attainment improved: the proportion with no education declined from 27.46 to 22.43%, while the proportion with higher education increased from 12.76 to 15.65%. Labor force participation also rose modestly, from 23.99 to 25.23%. The wealth distribution remained relatively stable across the two rounds, and a large majority of Muslim women continued to reside in rural areas, although the rural share increased slightly from 65.38 to 67.51%.

**Table 1:** Percentage distribution of socioeconomic characteristics of women aged 15–49 years in India based on NFHS-4 (2015–16) and NFHS-5 (2019–21).

**Socioeconomic characteristics**	**NFHS-4**	**NFHS-5**
Religion		
Hindu	80.57	81.36
Muslim	13.79	13.48
Christian	2.38	2.35
Sikh	1.66	1.57
Buddhist	0.92	0.63
Jain	0.18	0.23
Others	0.5	0.38
Age		
15–19	17.37	16.92
20–29	34.02	32.67
30–39	26.82	27.33
40–49	21.79	23.07
Caste		
SC/ST	29.55	31.18
OBC	43.42	42.92
Others	27.02	25.91
Place of residence		
Urban	34.62	32.49
Rural	65.38	67.51
Education		
No education	27.46	22.43
Primary	12.47	11.73
Secondary	47.31	50.18
Higher	12.76	15.65
Employment status		
Employed	23.99	25.23
Not Employed	76.01	74.77
Marital status		
Never married	22.73	23.76
Currently married	73.09	72
Formerly married	4.18	4.24
Wealth quintiles		
Poorest	17.73	18.5
Poorer	19.57	20
Middle	20.55	20.52
Richer	21.15	20.81
Richest	21	20.17
Region		
North	13.59	14.11
Central	23.65	24.89
East	22.11	22.76
West	14.37	14.09
South	22.76	20.45
North East	3.52	3.69

Author’s calculation based on NFHS-4 and NFHS-5 data. SC/ST = Scheduled Castes and Scheduled Tribes; OBC=Other Backward Castes; “Formerly married” includes women who are widowed, divorced, or separated at the time of the survey.

Table 2 reports hypertension prevalence and severity among Muslim women by socioeconomic characteristics. Overall hypertension prevalence increased slightly from 12.11% in NFHS-4 to 12.52% in NFHS-5. A strong age gradient is evident in both rounds. Prevalence was low among women aged 15–19 years, increasing only marginally from 3.27 to 3.43%, but was much higher among women aged 40–49 years, rising from 27.74 to 28.76%. Moderate hypertension was also concentrated in the oldest age group, increasing from 5.10 to 5.62% among women aged 40–49 years.

**Table 2:** Prevalence of hypertension (%) among Muslim women aged 15–49 years in India, by socioeconomic characteristics; NFHS-4 (2015–16) and NFHS-5 (2019–21).

**Socioeconomic characteristics**	**NFHS-4**				**NFHS-5**			
	**Hypertension** ^**1**^	**Mild** ^**2**^	**Moderate** ^**3**^	**Severe** ^**4**^	**Hypertension** ^**1**^	**Mild** ^**2**^	**Moderate** ^**3**^	**Severe** ^**4**^
All-India	12.11	8.39	1.77	0.71	12.52	8.98	1.94	0.71
Age								
15–19	3.27	1.97	0.29	0.13	3.43	2.09	0.2	0.27
20–29	6.61	4.48	0.53	0.3	6.46	4.54	0.69	0.22
30–39	15.79	11.48	2.33	0.93	14.93	11.23	2.08	0.81
40–49	27.74	19.01	5.1	1.88	28.76	20.47	5.62	1.89
Caste								
SC/ST	12.86	8.66	1.79	0.89	11.41	7.48	1.37	0.43
OBC	11.05	7.46	1.69	0.66	12.44	8.98	2.03	0.87
Others	13.06	9.25	1.85	0.75	12.68	9.1	1.91	0.6
Place of residence								
Urban	12.38	8.6	1.65	0.65	13.68	10.07	2.03	0.73
Rural	11.89	8.21	1.87	0.77	11.73	8.23	1.88	0.7
Education								
No education	16.1	11.11	2.65	1.19	18.36	13.24	3.22	1.21
Primary	13.46	9.71	1.97	0.61	14.41	10.58	2.13	0.85
Secondary	9.61	6.6	1.25	0.48	9.92	7.01	1.43	0.5
Higher	7.88	5.17	0.84	0.32	7.56	5.4	0.83	0.31
Employment status								
Employed	13.08	9.2	1.36	0.63	15.41	10.11	4.02	1.3
Not Employed	11.51	8.08	1.54	0.59	11.98	8.82	1.84	0.72
Marital status								
Never married	4.27	2.79	0.42	0.22	4.34	2.91	0.38	0.25
Currently married	14.62	10.17	2.2	0.86	14.88	10.7	2.37	0.83
Formerly married	20.14	14.31	3.28	1.35	24.17	18.15	4.48	1.73
Wealth quintiles								
Poorest	10.91	7.73	1.59	0.68	11.03	7.52	1.7	0.66
Poorer	11.51	8.21	1.8	0.78	11.31	7.96	1.88	0.69
Middle	12.33	8.24	1.8	0.88	12.84	9.32	2.22	0.8
Richer	13.02	9.02	1.97	0.66	13.62	9.9	1.97	0.72
Richest	12.37	8.5	1.61	0.56	13.69	10.11	1.91	0.71
Region								
North	13.39	8.63	1.75	0.73	11	7.81	1.14	0.4
Central	11.15	7.66	1.55	0.73	12.81	9.95	1.96	0.94
East	10.79	7.69	1.65	0.65	11.28	7.73	1.91	0.64
West	12.68	8.91	1.56	0.71	14.21	10.98	1.88	0.69
South	12.56	8.47	1.9	0.68	14.79	9.99	2.7	0.81
North East	17.03	12.72	3.23	1.04	11.6	7.66	1.72	0.66

Author’s calculation based on NFHS-4 and NFHS-5 data. SC/ST = Scheduled Castes and Scheduled Tribes; OBC=Other Backward Castes; “Formerly married” includes women who are widowed, divorced, or separated at the time of the survey. ^1^Hypertension was defined as Systolic Blood Pressure (SBP) ≥ 140 mmHg and/or Diastolic Blood Pressure (DBP) ≥ 90 mmHg, or current use of antihypertensive medication at the time of the survey. ^2^Mild hypertension: SBP 140–159 mmHg or DBP 90–99 mmHg. ^3^Moderate hypertension: SBP 160–179 mmHg or DBP 100–109 mmHg. ^4^Severe hypertension: SBP ≥180 mmHg or DBP ≥110 mmHg.

Educational differences show a clear inverse pattern. Hypertension prevalence was highest among Muslim women with no education, increasing from 16.10% in NFHS-4 to 18.36% in NFHS-5. In contrast, prevalence among women with higher education remained considerably lower, changing from 7.88 to 7.56%. Employment and marital status also show notable differences. Among employed Muslim women, hypertension prevalence increased from 13.08 to 15.41%, compared with 11.51 to 11.98% among non-employed women. Formerly married women had the highest prevalence among marital-status groups, increasing from 20.14 to 24.17%, compared with 4.27 to 4.34% among never-married women. However, the employment and marital-status results should be interpreted cautiously because the employment variable does not distinguish between different types of work, and the “formerly married” category combines widowed, divorced, and separated women.

The wealth gradient was not strictly linear. In NFHS-4, hypertension prevalence increased from 10.91% among the poorest women to 13.02% among the richer quintile, before declining slightly to 12.37% among the richest. In NFHS-5, prevalence increased from 11.03% among the poorest to 13.69% among the richest. These patterns suggest that hypertension among Muslim women is not confined to poorer groups and that wealth-related gradients may be flattening or shifting over time.

**Figure 2:**
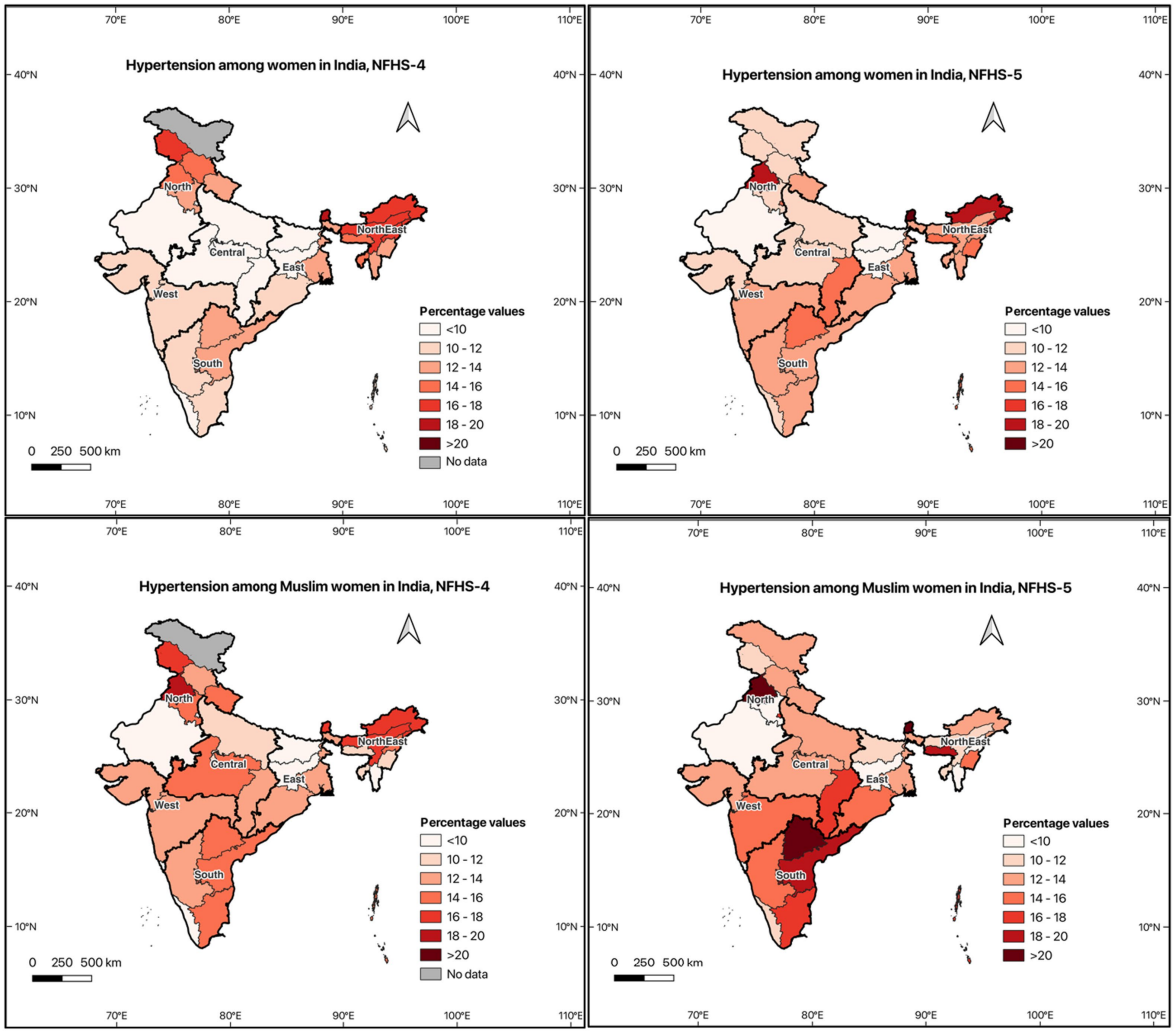
Region and State-level choropleth maps of hypertension prevalence (%) among women aged 15-49 years in India, NFHS-4 (2015-16) and NFHS-5 (2019-21). Administrative boundaries shown in the maps correspond to those recognized during the respective NFHS survey periods. In NFHS-4 (2015-16), Jammu and Kashmir and Ladakh were represented as a single administrative unit. In NFHS-5 (2019-21), Ladakh was delineated as a separate Union Territory and is depicted accordingly. Gray-shaded areas represent regions for which data were not available in the NFHS datasets.

Figure 2 presents the geographic distribution of hypertension prevalence among women and Muslim women across NFHS rounds. The maps show substantial spatial variation, but they should be interpreted as descriptive evidence of geographic heterogeneity rather than as formal evidence of socioeconomic inequality. District-level choropleth maps are provided in Supplementary File S1.

### 5.2 Socioeconomic inequality in hypertension

**Figure 3:**
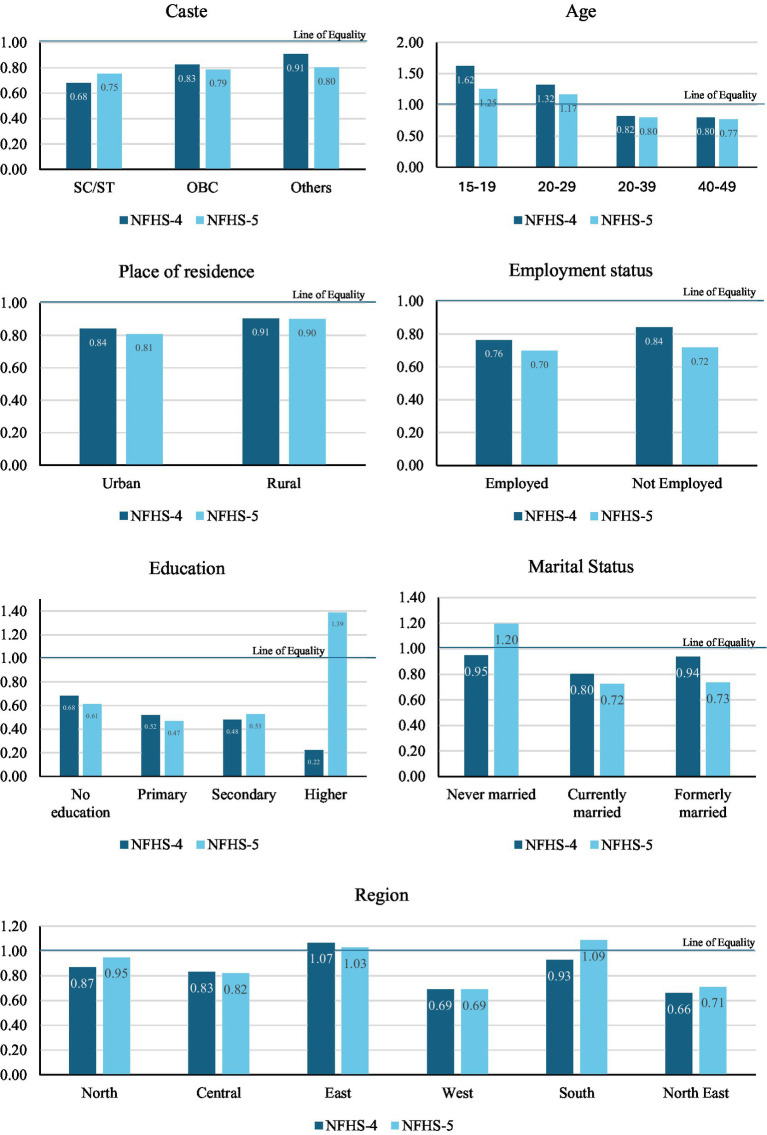
Poor-to-rich ratios for hypertension prevalence across socioeconomic subgroups among Muslim women in India, NFHS-4 (2015–16) and NFHS-5 (2019–21).

Figure 3 presents Poor-Rich ratios for hypertension prevalence among Muslim women across the two NFHS rounds. Several PR ratios moved closer to unity, suggesting a narrowing of the poorest-richest gap across socioeconomic subgroups. A particularly notable change is observed among highly educated Muslim women. The PR ratio shifted from 0.22 in NFHS-4 to 1.39 in NFHS-5. Since the PR ratio is defined as hypertension prevalence among women in the poorest wealth quintile divided by prevalence among women in the richest wealth quintile, this indicates a reversal in the poorest-richest contrast within the higher-educated subgroup: from substantially lower prevalence among the poorest relative to the richest in NFHS-4 to higher prevalence among the poorest relative to the richest in NFHS-5. The table version of the PR ratios is provided in Supplementary File S2.

Table 3 reports Concentration Index estimates for hypertension among Muslim women. CI values remained positive for most subgroups, indicating that hypertension continued to be relatively concentrated among better-off women. For example, among women with secondary education, the CI increased from 0.104 in NFHS-4 to 0.125 in NFHS-5, while among employed women it increased from 0.042 to 0.123. Among currently married women, the CI increased from 0.044 to 0.067, and among formerly married women, it increased from 0.019 to 0.062. Regionally, positive CI values persisted in the North, Central, West, and North-East, while the South showed negative CI values in both survey rounds.

**Table 3:** Trends in socioeconomic inequalities in hypertension among Muslim women in India using the Concentration Index (CI) evidence from NFHS-4 (2015–16) and NFHS-5 (2019–21).

**Socioeconomic characteristics**	**NFHS-4**		**NFHS-5**	
	**CI**	**SE**	**CI**	**SE**
Caste				
SC/ST	0.054***	0.017	0.057***	0.019
OBC	0.033***	0.008	0.044***	0.008
Others	0.031***	0.007	0.052***	0.007
Age				
15–19	−0.087***	0.022	−0.04**	0.023
20–29	−0.038***	0.012	−0.023**	0.012
30–39	0.04***	0.009	0.04***	0.009
40–49	0.038***	0.007	0.059***	0.007
Place of residence				
Urban	0.010	0.008	0.02**	0.008
Rural	0.036***	0.006	0.036***	0.006
Education				
No education	0.084***	0.007	0.097***	0.008
Primary	0.117***	0.013	0.141***	0.013
Secondary	0.104***	0.008	0.125***	0.008
Higher	0.017	0.021	0.04**	0.020
Employment status				
Employed	0.042	0.028	0.123***	0.029
Not Employed	0.019	0.013	0.058***	0.015
Marital status				
Never married	0.015	0.02	−0.003	0.017
Currently married	0.044***	0.01	0.067***	0.006
Formerly married	0.019	0.02	0.062***	0.019
Region				
North	0.015**	0.009	0.036***	0.010
Central	0.036***	0.010	0.039***	0.011
East	0.037***	0.013	0.012	0.013
West	0.075***	0.020	0.064***	0.016
South	−0.058***	0.014	−0.033***	0.012
North East	0.059***	0.011	0.076***	0.013

Author’s calculation based on NFHS-4 and NFHS-5 data. SC/ST = Scheduled Castes and Scheduled Tribes; OBC=Other Backward Classes; “Formerly married” includes women who are widowed, divorced, or separated at the time of the survey; CI is the Concentration index based on the different socio-economic factors against the prevalence of women’s hypertension; SE = Standard Error. *p < 0.05; **p < 0.01; ***p < 0.001.

The PR ratio and CI results should be interpreted as complementary rather than contradictory. The PR ratio compares only the two extremes of the wealth distribution, namely the poorest and richest quintiles. In contrast, the CI summarizes inequality across the full wealth distribution. A positive CI can therefore coexist with a PR ratio close to or below one if hypertension prevalence is higher among middle or richer quintiles than among one or both extremes. This pattern is visible in the descriptive estimates, particularly in NFHS-4, where prevalence increased from the poorest quintile to the richest quintile and then declined slightly among the richest. The evidence points to gradient flattening, non-linearity, and partial reversal rather than a simple linear poor-rich gradient.

### 5.3 Multivariable regression results

Table 4 presents adjusted odds ratios for hypertension among Muslim women from separate multivariable logistic regression models estimated for NFHS-4 and NFHS-5. These models account simultaneously for age, education, wealth, employment status, marital status, caste, place of residence, and region.

**Table 4:** Odds ratios for hypertension among Muslim women in India across socioeconomic factors: NFHS-4 (2015–16) and NFHS-5 (2019–21).

**Characteristics**	**NFHS-4**			**NFHS-5**		
	**Odds ratio**	**SE**	**[95% conf. interval]**	**Odds ratio**	**SE**	**[95% conf. interval]**
Caste (SC/ST ®)						
OBC	0.881	0.081	[0.735–1.055]	0.997	0.115	[0.795–1.250]
Others	1.126	0.097	[0.952–1.333]	1.144	0.132	[0.912–1.433]
Age (15–19®)						
20–29	1.696***	0.199	[1.347–2.136]	2.054***	0.297	[1.546–2.727]
30–39	4.085***	0.525	[3.176–5.254]	4.094***	0.637	[3.017–5.555]
40–49	9.194***	1.199	[7.121–11.871]	8.334***	1.315	[6.117–11.355]
Residence (Urban ®)						
Rural	0.94	0.053	[0.841–1.050]	0.932	0.064	[0.815–1.066]
Education (no education ®)						
Primary	1.08	0.084	[0.928–1.257]	0.906	0.081	[0.760–1.080]
Secondary	0.96	0.062	[0.846–1.089]	0.891	0.066	[0.771–1.030]
Higher	0.862	0.100	[0.688–1.081]	0.645***	0.087	[0.496–0.840]
Employment (currently employed®)						
Not Employed	0.926	0.060	[0.816–1.053]	0.949	0.071	[0.820–1.099]
Marital status (never married®)						
Currently married	1.177**	0.113	[0.975–1.421]	1.151	0.131	[0.921–1.438]
Formerly married	1.272**	0.183	[0.960–1.687]	1.36**	0.229	[0.978–1.892]
Wealth (Poorest ®)						
Second quintile	1.13	0.098	[0.954–1.338]	1.178**	0.113	[0.976–1.421]
Middle quintile	1.139	0.103	[0.954–1.360]	1.214**	0.125	[0.992–1.484]
Fourth quintile	1.137	0.110	[0.941–1.373]	1.507***	0.163	[1.219–1.862]
Highest quintile	1.102	0.118	[0.894–1.360]	1.365**	0.169	[1.070–1.740]
Regions (North ®)						
Central	0.783***	0.057	[0.678–0.903]	1.226**	0.109	[1.029–1.460]
East	0.75***	0.064	[0.635–0.886]	1.118	0.106	[0.928–1.346]
West	0.73***	0.080	[0.589–0.905]	1.183	0.130	[0.953–1.468]
South	0.928	0.078	[0.787–1.094]	1.342***	0.130	[1.111–1.622]
North East	1.163**	0.102	[0.980–1.380]	1.28**	0.133	[1.045–1.568]
_cons	0.042***	0.007	[0.031–0.058]	0.027***	0.005	[0.018–0.040]

Author’s calculation based on NFHS-4 and NFHS-5 data. Note(s): SC/ST = Scheduled castes and Scheduled Tribes; OBC=Other Backward Classes; hypertension was defined as systolic blood pressure ≥140 mmHg and/or diastolic blood pressure ≥90 mmHg, or current use of antihypertensive medication at the time of the survey. “Formerly married” includes women who are widowed, divorced, or separated at the time of the survey: 95% conf. Interval = 95% confidence interval; SE = Standard Error. Reference categories: SC/ST; 15–19 years; urban; no education; poorest quintile; North. ORs are interpreted relative to these baselines. _cons reflects the baseline odds of hypertension for the reference group. *p* < 0.05; *p* < 0.01; *p* < 0.001. Odds ratios (OR) with 95% confidence intervals are estimated using survey-weighted multivariable logistic regression, accounting for complex survey design. Reference categories for each variable are as follows: Caste: SC/ST; Age: 15–19 years; Residence: Urban; Education: No education; Employment: Currently employed; Marital status: Never married; Wealth quintile: Poorest; Region: North. *p < 0.05; **p < 0.01; ***p < 0.001.

Table 4 presents adjusted odds ratios for hypertension among Muslim women from separate survey-weighted logistic regression models for NFHS-4 and NFHS-5. The models adjust for age, caste, residence, education, employment status, marital status, wealth quintile, and region.

Age remained the strongest predictor of hypertension in both survey rounds. Compared with women aged 15–19 years, the odds of hypertension increased sharply among women aged 30–39 years and 40–49 years. In NFHS-4, women aged 30–39 years had 4.09 times higher odds of hypertension, while women aged 40–49 years had 9.19 times higher odds. A similar pattern was observed in NFHS-5, with corresponding odds ratios of 4.09 and 8.33. This confirms a steep age gradient in hypertension risk among Muslim women.

Educational differences were modest after adjustment. In NFHS-4, education categories did not show statistically clear differences relative to women with no education. In NFHS-5, however, higher education was associated with lower odds of hypertension (OR = 0.65; 95% CI: 0.50–0.84), suggesting that the protective association of education was concentrated among women with higher education in the more recent survey round.

The adjusted wealth gradient changed notably between the two survey rounds. In NFHS-4, none of the wealth quintiles differed significantly from the poorest quintile, suggesting the absence of a clear adjusted wealth gradient in hypertension among Muslim women in 2015–16. By NFHS-5, however, the association became positive and statistically significant among women in the upper wealth groups. Compared with women in the poorest quintile, those in the fourth wealth quintile had higher odds of hypertension (OR = 1.51; 95% CI: 1.22–1.86), and women in the richest quintile also showed higher odds (OR = 1.37; 95% CI: 1.07–1.74). This shift indicates that hypertension risk among Muslim women was no longer concentrated only among poorer groups; rather, by NFHS-5, relatively better-off women also showed elevated adjusted odds of hypertension. This finding reinforces the broader evidence of gradient flattening and partial reversal observed in the descriptive and inequality analyses.

Caste and rural–urban residence showed weaker associations after adjustment. OBC and other-caste Muslim women did not differ significantly from SC/ST Muslim women, and rural residence was not significantly associated with hypertension after controlling for other characteristics. Employment status also requires cautious interpretation. Relative to currently employed women, not-employed women had slightly lower odds of hypertension in both NFHS-4 and NFHS-5, but the differences were not statistically significant. Since the binary employment variable does not distinguish between formal salaried work, casual labor, self-employment, agricultural work, home-based work, or unpaid family work, this result should be interpreted as a broad association with labor force participation rather than as evidence about specific occupational conditions.

Marital status showed some evidence of elevated risk among formerly married women, especially in NFHS-5 (OR = 1.36; 95% CI: 0.98–1.89). However, this category combines widowed, divorced, and separated women, and should therefore be interpreted as a broad marker of marital disruption rather than as evidence specific to any one marital state. Regional differences also persisted after adjustment. In NFHS-5, Muslim women in the Central (OR = 1.23; 95% CI: 1.03–1.46), Southern (OR = 1.34; 95% CI: 1.11–1.62), and North-Eastern regions (OR = 1.28; 95% CI: 1.05–1.57) had higher odds of hypertension than those in the Northern region.

The odds-ratio forest plots are provided in Supplementary File S3.

### 5.4 Spatial inequality and clustering

**Figure 4:**
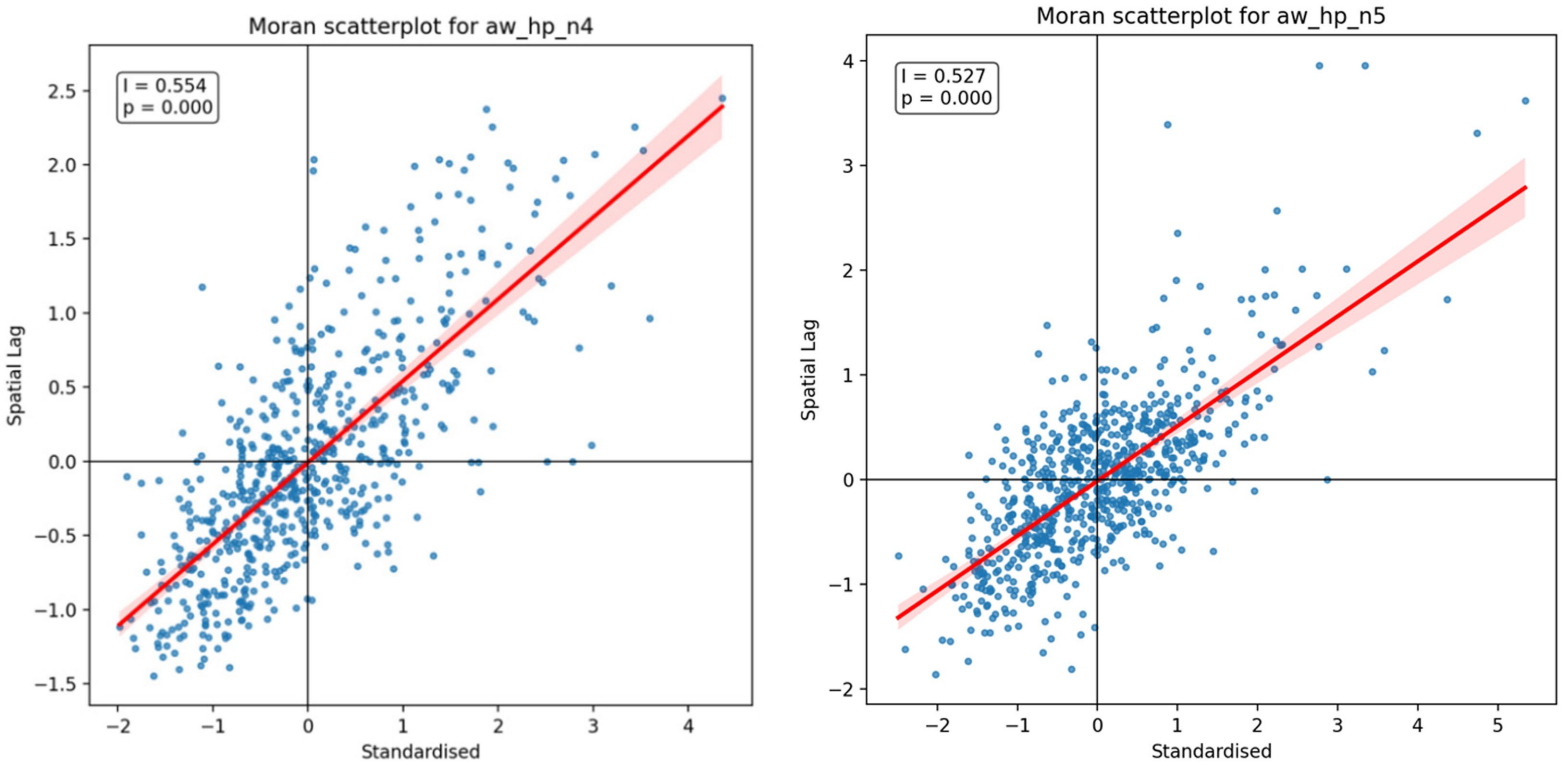
Moran’s *I* scatterplots of district-level hypertension prevalence among women aged 15–49 years in India, NFHS-4 (2015–16) and NFHS-5 (2019–21). Author’s calculation based on NFHS-4 and NFHS-5. Moran’s *I* statistics and corresponding p-values are reported within each panel. The scatterplots show the relationship between district-level hypertension prevalence and the spatially lagged mean of neighboring districts, based on a first-order contiguity spatial weights matrix. Each point represents a district.

**Figure 5:**
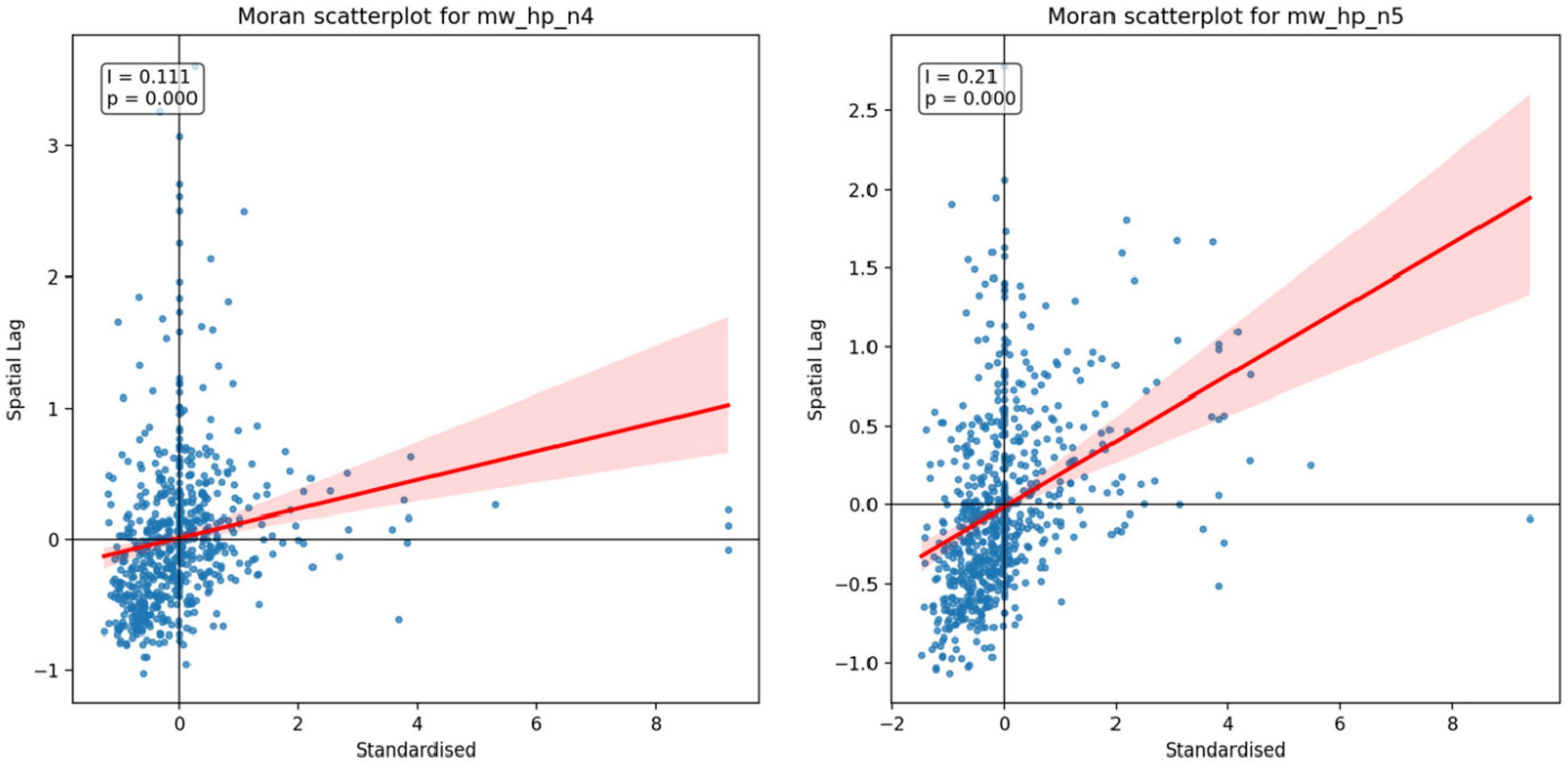
Moran’s *I* scatterplots of district-level hypertension prevalence among Muslim women aged 15–49 years in India, NFHS-4 (2015–16) and NFHS-5 (2019–21). Author’s calculation based on NFHS-4 and NFHS-5. Moran’s I statistics and corresponding p-values are reported within each panel. The scatterplots show the relationship between district-level hypertension prevalence and the spatially lagged mean of neighboring districts, based on a first-order contiguity spatial weights matrix. Each point represents a district.

Figures 4, 5 report Moran’s *I* scatterplots for district-level hypertension prevalence among all women and Muslim women, respectively. In both NFHS-4 and NFHS-5, Moran’s *I* values were positive and statistically significant, indicating non-random spatial clustering of hypertension prevalence across districts. Among Muslim women, Moran’s *I* increased from 0.111 in NFHS-4 to 0.210 in NFHS-5, suggesting that district-level hypertension prevalence became more spatially clustered over time.

**Figure 6:**
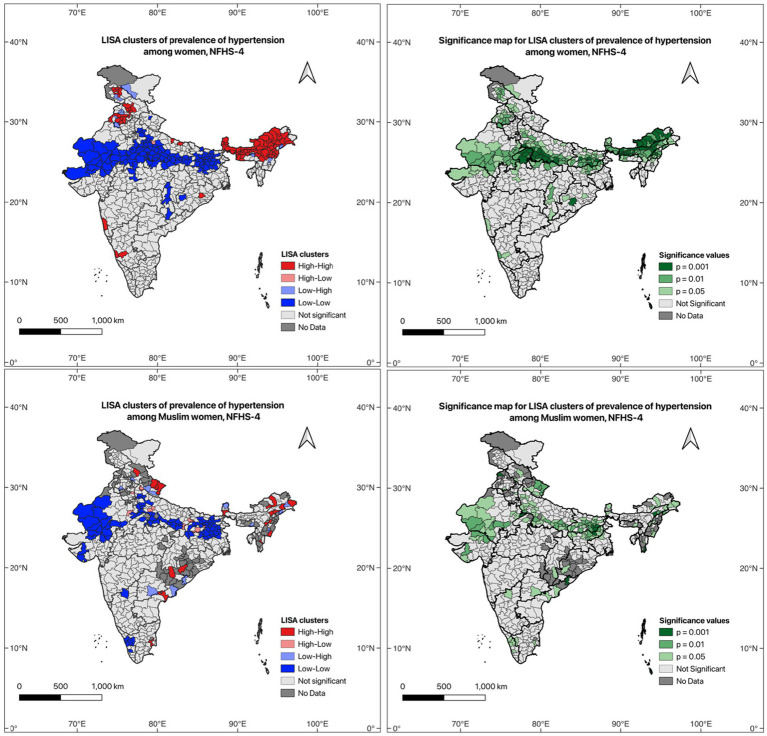
Local indicators of spatial association (LISA) clusters for hypertension prevalence among all women and Muslim women aged 15–49 years in India, NFHS-4 (2015-16). Source: Authors’ calculation based on NFHS-4 data. District boundaries reflect the administrative divisions in place during the respective NFHS survey periods. Dark gray-shaded areas represent districts for which NFHS did not report hypertension estimates, either due to non coverage, administrative exclusion, or insufficient sample size for reliable prevalence reporting. District boundaries reflect the administrative divisions in place during the respective NFHS survey periods. Grey-shaded districts represent areas for which NFHS did not report hypertension estimates, either due to non-coverage, administrative exclusion, or insufficient sample size for reliable prevalence reporting. Since the analysis is restricted to Muslim women at the district level, some included districts may still have relatively small subsamples. LISA classifications should therefore be interpreted as indicative of broad spatial clustering patterns rather than precise district-level rankings.

**Figure 7:**
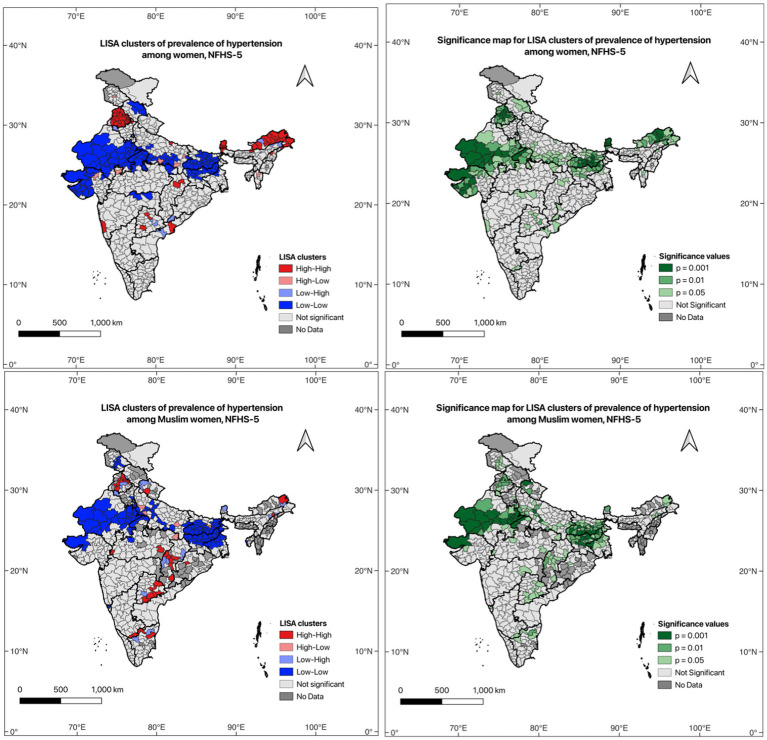
Local Spatial Indicators Association (LISA) using Moran’s I for hypertension prevalence among all women and Muslim women aged 15-49 years in India, NFHS-5 (2019-21). Source: Authors’ calculation based on NFHS-5 data. Note: District boundaries reflect the administrative divisions in place during the respective NFHS survey periods. Dark grey-shaded areas represent districts for which NFHS did not report hypertension estimates, either due to non-coverage, administrative exclusion, or insufficient sample size for reliable prevalence reporting. District boundaries reflect the administrative divisions in place during the respective NFHS survey periods. Grey-shaded districts represent areas for which NFHS did not report hypertension estimates, either due to non-coverage, administrative exclusion, or insufficient sample size for reliable prevalence reporting. Since the analysis is restricted to Muslim women at the district level, some included districts may still have relatively small subsamples. LISA classifications should therefore be interpreted as indicative of broad spatial clustering patterns rather than precise district-level rankings.

Figures 6, 7 present Local Indicators of Spatial Association (LISA) cluster maps for NFHS-4 and NFHS-5, respectively. The LISA results identify localized high-high and low-low clusters of hypertension prevalence. These clusters suggest that hypertension risk among Muslim women is geographically patterned rather than randomly distributed across districts. The spatial clustering among Muslim women also differs in location and intensity from the patterns observed among all women, indicating the value of group-specific spatial analysis.

These spatial findings should be interpreted with caution. Since the district-level analysis is restricted to Muslim women, some district estimates may be based on relatively small unweighted denominators. Although districts with unavailable or insufficient estimates were excluded and displayed in grey, the remaining district-level prevalence estimates may still vary in statistical precision. The LISA results are therefore best understood as evidence of broad spatial clustering rather than as exact district-level rankings of hypertension risk.

## 6 Discussion

### 6.1 Hypertension, epidemiological transition, and gradient dynamics

The findings of this study situate hypertension among Muslim women in India within the broader dynamics of epidemiological transition, while also showing that the socioeconomic distribution of hypertension does not follow a simple deprivation-based pattern. Classical accounts of epidemiological transition suggest that, as societies undergo demographic and economic change, the burden of disease shifts from infectious to chronic conditions, with non-communicable diseases often becoming more visible among socioeconomically advantaged groups before diffusing more widely across the population (2, 4, 5). The age-specific patterns observed in this study, particularly the sharp increase in hypertension prevalence after age 30, are consistent with India’s movement into a chronic disease regime.

At the same time, the socioeconomic distribution of hypertension among Muslim women appears more complex than a conventional poor-rich gradient. Evidence from prevalence estimates, Poor-Rich ratios, Concentration Indices, and adjusted regression models points to gradient flattening and, in some subgroups, partial reversal. Hypertension is not confined to poorer or less educated women; elevated prevalence is also observed among wealthier, urban, and employed Muslim women, especially in NFHS-5. These patterns are consistent with economic explanations of gradient dynamics, where exposure to transition-related risks such as sedentary work, dietary change, psychosocial stress, and urban living may emerge before protective resources and preventive care are evenly distributed (12, 35). However, these mechanisms should be interpreted as plausible explanations rather than directly observed pathways, since the NFHS does not measure all relevant behavioral, occupational, or psychosocial exposures.

The reversal in the PR ratio among higher-educated Muslim women further illustrates that socioeconomic gradients are not uniform across subgroups. Rather, the direction and magnitude of wealth-related differences appear to vary by education and survey round. The persistence of positive Concentration Indices alongside PR ratios approaching unity also suggests that inequality is neither simply widening nor fully reversing. Instead, the results point to a transitional phase in which hypertension risk is increasingly distributed across socioeconomic strata. The regression results strengthen this interpretation: while wealth quintiles were not significantly associated with hypertension in NFHS-4, the fourth and richest quintiles showed significantly higher adjusted odds in NFHS-5. This suggests that the wealth gradient became more positive over time among Muslim women, although the evidence should be understood as associational rather than causal. This dynamic perspective aligns with arguments that health gradients evolve with development and should be analyzed as temporal and distributional processes rather than fixed relationships (2, 36).

### 6.2 Socioeconomic health gradients and minority-status-linked pathways

A central contribution of this study is that it documents how hypertension is socially patterned within Muslim women, a religious minority population that remains underrepresented in non-communicable disease research in India. The findings do not demonstrate that religion causally modifies hypertension gradients relative to other religious groups, because the analysis is designed as a within-group study rather than an inter-religious comparison. Instead, the results show that among Muslim women, hypertension is associated with age, wealth, education, employment, marital status, and region in ways that are consistent with minority-status-linked structural pathways.

The observed association between higher wealth and hypertension in NFHS-5 suggests that socioeconomic advancement may not uniformly translate into lower cardiovascular risk among Muslim women. This does not imply that wealth itself causes hypertension. A more cautious interpretation is that relatively better-off women may be more exposed to transition-related risk environments, including sedentary work, urban lifestyles, dietary change, or better detection through healthcare contact. Similarly, higher education appears protective in NFHS-5, but the broader wealth pattern remains non-linear. Taken together, these findings suggest that socioeconomic position may operate through multiple and sometimes competing pathways.

Employment status also requires careful interpretation. The descriptive results show higher hypertension prevalence among employed Muslim women, but the adjusted models do not provide strong evidence of an independent employment effect. Moreover, the NFHS employment variable does not distinguish between formal salaried work, casual labor, agricultural work, self-employment, home-based work, or unpaid family work. These forms of work may differ substantially in income security, autonomy, physical demand, time pressure, and exposure to work-related stress. Prior evidence suggests that precarious employment and job strain may be associated with adverse health outcomes, including elevated blood pressure and cardiovascular risk (37, 38). In the present study, however, occupational stress, job insecurity, and employment quality cannot be directly tested. The employment findings should therefore be interpreted as broad associations with labor force participation rather than as evidence that employment itself increases hypertension risk.

Marital status shows another important but heterogeneous association. Formerly married Muslim women show higher odds of hypertension than never-married women, suggesting that marital disruption may be associated with heightened vulnerability. This interpretation should remain cautious. The “formerly married” category combines widowed, divorced, and separated women, although these marital states may involve different social and economic circumstances. Widowhood may be linked to bereavement and loss of household support, while divorce and separation may involve different forms of stigma, household restructuring, or economic insecurity. Since these categories were not analyzed separately, the result should be understood as a broad association between marital disruption and hypertension risk, not as evidence specific to widowhood, divorce, or separation.

The findings support the analytical value of examining health gradients within minority populations. They suggest that among Muslim women, socioeconomic resources, employment, marital status, and regional context are associated with hypertension in ways that may reflect unequal exposure to risk and uneven access to protective resources. However, the NFHS data do not directly measure discrimination, bargaining power, work quality, psychosocial stress, dietary intake, physical activity, or quality of healthcare access. These should therefore be understood as theoretically plausible mechanisms rather than confirmed empirical pathways.

### 6.3 Spatial inequality, institutions, and uneven transitions

The spatial analysis shows that hypertension among Muslim women is geographically patterned rather than randomly distributed. Positive and statistically significant Moran’s *I* values, together with the LISA clusters, indicate spatial clustering of district-level hypertension prevalence. These findings are consistent with the idea that chronic disease risk unfolds unevenly across regions, reflecting differences in urbanization, health-system reach, socioeconomic development, and local institutional contexts.

However, the spatial results should not be over-interpreted as direct evidence of specific institutional or political-economic mechanisms. The NFHS does not directly measure district-level institutional capacity, quality of primary healthcare, screening intensity, public spending, or discrimination in access to services. Spatial clustering may reflect several overlapping processes, including differences in healthcare detection, urbanization, lifestyle exposure, demographic composition, and local socioeconomic conditions. Therefore, references to institutional capacity or regional political economy are best understood as plausible contextual explanations rather than mechanisms directly tested in the analysis.

A further limitation concerns the district-level spatial analysis. Because the LISA analysis for Muslim women is based on district-level subsamples, some prevalence estimates may be less stable where the number of Muslim women sampled is small. Although districts with unavailable or insufficient estimates were excluded from the maps, the remaining district-level estimates may still differ in precision. For this reason, the spatial findings should be interpreted as broad evidence of clustering rather than as precise district-level rankings. The analysis also does not apply spatial Empirical Bayes smoothing, which could stabilize small-area rates by reducing the influence of sparse denominators (39). Future work focused on small-area estimation could extend this analysis using Empirical Bayes or related smoothing methods.

Despite these limitations, the spatial findings remain useful. They show that aggregate national estimates can obscure subnational heterogeneity and that hypertension risk among Muslim women has a geographic dimension. Group-sensitive spatial analysis can therefore complement conventional socioeconomic inequality measures by identifying areas where screening, prevention, and follow-up may require closer attention.

### 6.4 Policy implications and contributions

The results have implications for hypertension screening and prevention. First, the findings caution against assuming that hypertension risk among Muslim women is concentrated only among the poorest. The emergence of elevated risk among relatively better-off groups in NFHS-5 suggests that screening strategies should not be limited to economically deprived women alone. At the same time, this should not be interpreted as a reason to reduce attention to poorer women, who may face weaker access to diagnosis, treatment, and long-term disease management.

Second, the findings suggest that employment and marital status may be useful markers for identifying women who require closer attention in hypertension prevention programs. However, because the NFHS does not directly measure work quality, psychosocial stress, household bargaining, or social support, policy interpretations should remain cautious. Rather than concluding that employment or marital disruption directly causes hypertension, the results suggest that women’s social and economic circumstances may shape exposure to risk and access to care.

Third, the spatial clustering of hypertension indicates that prevention strategies should account for subnational heterogeneity. Districts and regions with persistent clustering may require stronger screening, referral, and follow-up systems. However, given the limitations of district-level subsamples for Muslim women, the spatial findings should be treated as signals for further investigation rather than definitive district rankings.

From a scholarly perspective, this paper makes three contributions. First, it provides nationally representative evidence on hypertension among Muslim women in India, a population that remains underrepresented in non-communicable disease research. Second, it shows that socioeconomic gradients in hypertension among Muslim women are dynamic, non-linear, and partly shifting over time. Third, it integrates inequality measures, regression analysis, and spatial methods to examine both social and geographic dimensions of hypertension risk. These contributions support a more disaggregated approach to studying health inequality during epidemiological transition, while also highlighting the need for future research that can directly test the occupational, household, healthcare, and institutional mechanisms suggested by the findings.

## 7 Conclusion

This study examined trends and socioeconomic-spatial inequalities in hypertension among Muslim women aged 15–49 years in India using NFHS-4 and NFHS-5 data. By combining nationally representative survey data with inequality measures, survey-weighted multivariable regression, and spatial analysis, the paper assessed whether hypertension among Muslim women follows a simple deprivation gradient or a more complex pattern of flattening, non-linearity, and partial reversal. This framing is consistent with epidemiological transition theory and population-economic approaches that view health gradients as dynamic rather than fixed relationships (2, 4, 5, 12).

The findings suggest that hypertension among Muslim women cannot be understood solely as a disease of poverty. Overall prevalence increased slightly between NFHS-4 and NFHS-5, and a strong age gradient persisted across both survey rounds. At the same time, wealth-related patterns were not strictly linear. In NFHS-4, wealth quintiles were not significantly associated with hypertension after adjustment, whereas in NFHS-5, women in the fourth and richest wealth quintiles had significantly higher odds than women in the poorest quintile. The PR-ratio reversal among higher-educated Muslim women and the persistence of positive Concentration Indices further suggest that socioeconomic gradients are changing rather than stable. These patterns align with earlier arguments that chronic disease risks may initially appear among relatively advantaged groups before diffusing more widely across the population (11, 40).

The spatial results also show that hypertension among Muslim women is geographically patterned. Moran’s I and LISA findings indicate non-random clustering of district-level hypertension prevalence, suggesting that chronic disease risk is shaped not only by individual socioeconomic position but also by place. This is consistent with spatial approaches that emphasize uneven development, regional heterogeneity, and local clustering in health outcomes (20, 33, 34). However, because district-level estimates for Muslim women may be less stable where subsamples are small, these spatial findings should be interpreted as broad clustering tendencies rather than precise district-level rankings. Future work could use small-area estimation or Empirical Bayes smoothing to improve the stability of district-level estimates in sparse populations (39).

A key contribution of this paper is its within-group focus on Muslim women, a population often underrepresented in non-communicable disease research in India. The findings are consistent with minority-status-linked structural pathways, but they do not directly test whether religion modifies hypertension gradients relative to other religious groups. Similarly, mechanisms such as occupational stress, household bargaining, marital vulnerability, healthcare access, and institutional capacity remain plausible explanations rather than directly measured channels in NFHS data. This cautious interpretation is important because religion is treated here not as a biological or cultural cause of hypertension, but as a social location that may intersect with education, employment, wealth, marital status, and region (7–10).

From a policy perspective, the findings caution against assuming that hypertension risk is concentrated only among the poorest women. Screening and prevention strategies should account for age, education, wealth, marital status, employment status, and regional context, while also ensuring that minority women are not overlooked in non-communicable disease prevention. At the same time, the results should not be read as implying that employment, marital disruption, or minority status directly cause hypertension. Rather, they point to the need for more disaggregated public health strategies and future research with richer measures of work conditions, household resources, healthcare access, and local institutional capacity. Inter-religious comparative designs and longitudinal data would be especially useful for testing whether socioeconomic gradients in hypertension differ across religious groups and how these gradients evolve over the life course.

## Data Availability

Publicly available datasets were analyzed in this study. This data can be found here: https://www.dhsprogram.com/data/dataset_admin.

## Supplementary material

SUPPLEMENTARY FILE S1 District-level choropleth maps of hypertension prevalence (%) among all women and Muslim women aged 15–49 years in India, NFHS-4 (2015–16) and NFHS-5 (2019–21).

SUPPLEMENTARY FILE S2 Poor-to-Rich ratios for hypertension prevalence across socioeconomic subgroups among Muslim women in India, NFHS-4 (2015–16) and NFHS-5 (2019–21).

SUPPLEMENTARY FILE S3 Adjusted odds-ratio forest plots for hypertension among Muslim women in India, NFHS-4 (2015–16) and NFHS-5 (2019–21).
