# Supplementary material for: Trends and socioeconomic-spatial inequalities in hypertension among Muslim women in India, 2015–2021: evidence from the National Family Health Surveys
Source: Front Public Health. 2026 Jun 11;14:1828079. doi: 10.3389/fpubh.2026.1828079 (PMC13294860; doi:10.3389/fpubh.2026.1828079)
Supplement: SUPPLEMENTARY FILE S1 — District-level choropleth maps of hypertension prevalence (%) among all women and Muslim women aged 15–49 years in India, NFHS-4 (2015–16) and NFHS-5 (2019–21). [file Supplementary_file_1.docx]

**S1: District-level choropleth maps of hypertension prevalence (%) among all women and Muslim women aged 15-49 years in India, NFHS-4 (2015-16) and NFHS-5 (2019-21).**


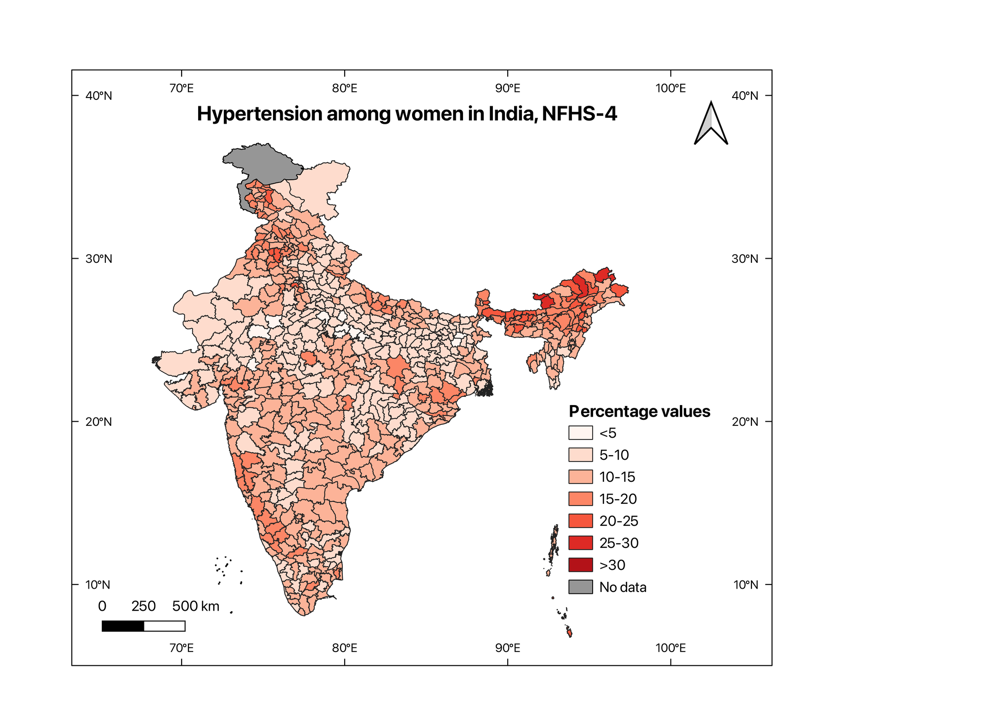

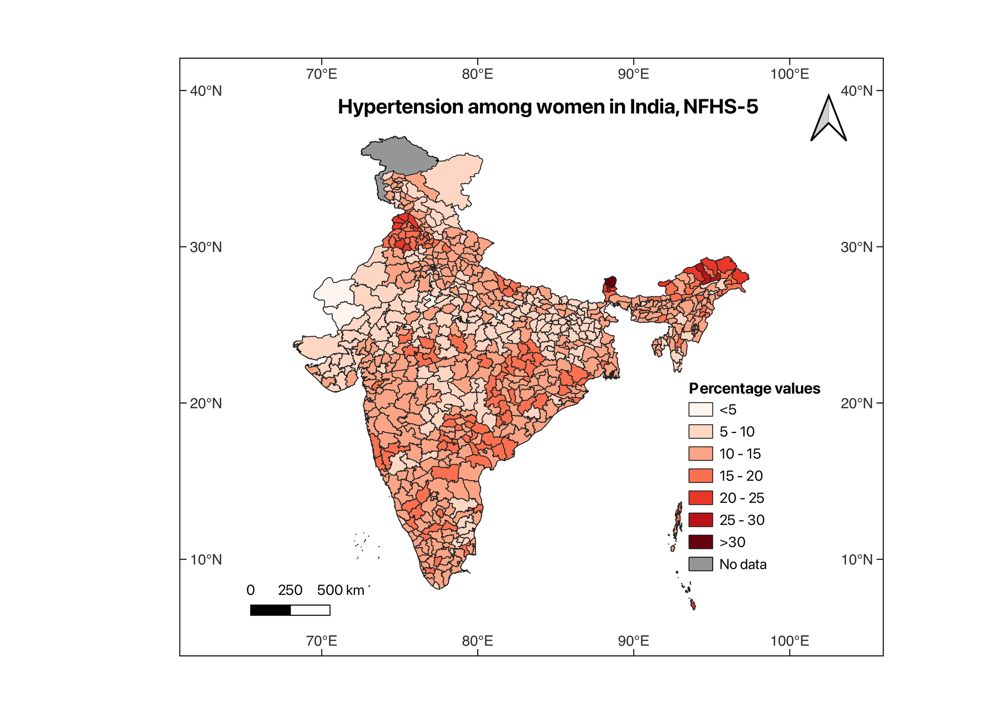

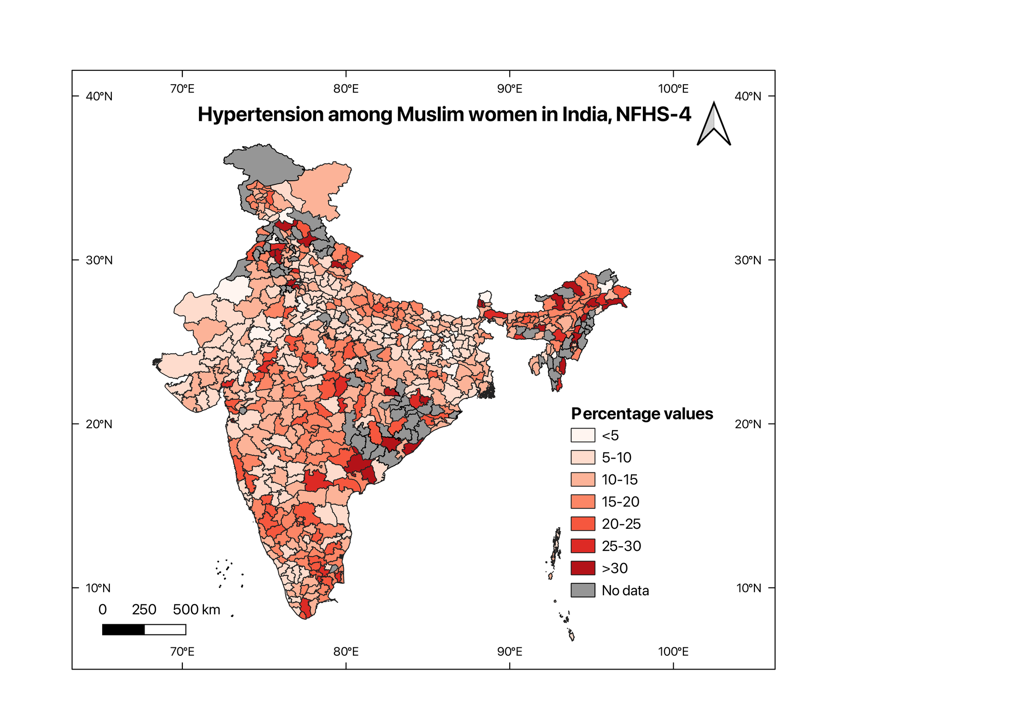

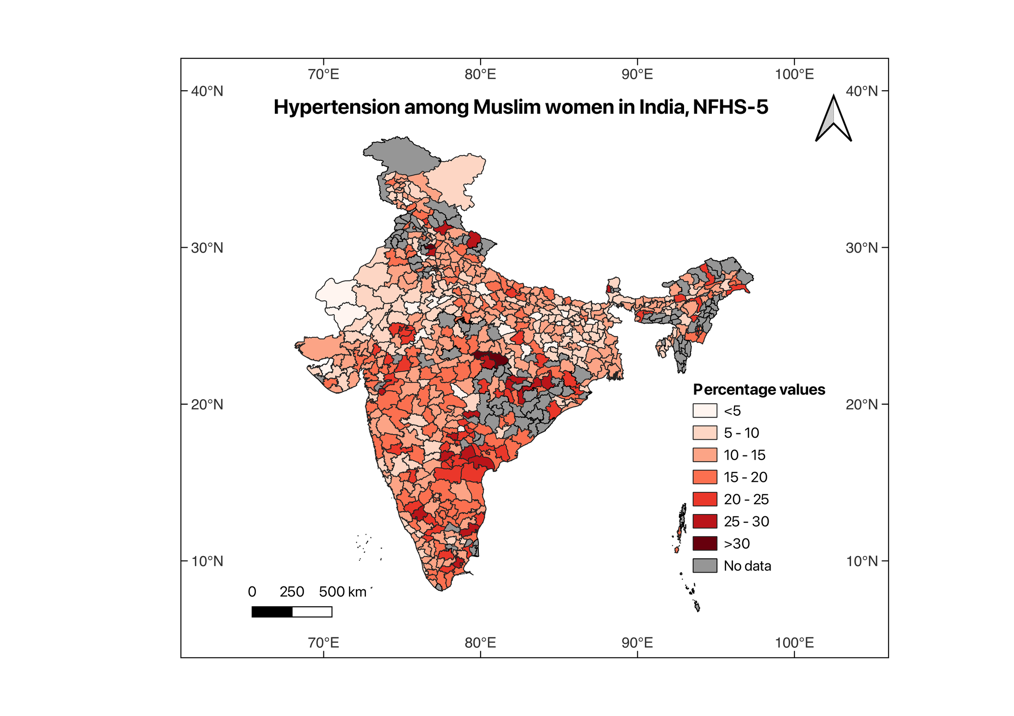


**Note:** District boundaries reflect the administrative divisions in place during the respective NFHS survey periods. In NFHS-4 (2015-16), Jammu & Kashmir and Ladakh were represented as a single state, whereas in NFHS-5 (2019-21), Ladakh was delineated as a separate Union Territory following its administrative reorganisation in 2019. Grey-shaded areas represent districts for which NFHS did not report hypertension estimates, either due to non-coverage, administrative exclusion, or insufficient sample size for reliable prevalence reporting.
