# Supplementary material for: Trends and socioeconomic-spatial inequalities in hypertension among Muslim women in India, 2015–2021: evidence from the National Family Health Surveys
Source: Front Public Health. 2026 Jun 11;14:1828079. doi: 10.3389/fpubh.2026.1828079 (PMC13294860; doi:10.3389/fpubh.2026.1828079)
Supplement: SUPPLEMENTARY FILE S2 — Poor-to-Rich ratios for hypertension prevalence across socioeconomic subgroups among Muslim women in India, NFHS-4 (2015–16) and NFHS-5 (2019–21). [file Supplementary_file_2.docx]

**S2** : **Poor-to-Rich (P-R) ratios for hypertension prevalence by wealth quintile among Muslim women in India from NFHS-4 (2015-16) and NFHS-5 (2019-21).**

| **Caste** | **NFHS-4** | **NFHS-5** |
| --- | --- | --- |
| SC/ST | 0.68 | 0.75 |
| OBC | 0.83 | 0.79 |
| Others | 0.91 | 0.80 |
| **Age** |  |  |
| 15-19 | 1.62 | 1.25 |
| 20-29 | 1.32 | 1.17 |
| 20-39 | 0.82 | 0.80 |
| 40-49 | 0.80 | 0.77 |
| **Place of Residence** |  |  |
| Urban | 0.84 | 0.81 |
| Rural | 0.91 | 0.90 |
| **Education** |  |  |
| No education | 0.68 | 0.61 |
| Primary | 0.52 | 0.47 |
| Secondary | 0.48 | 0.53 |
| Higher | 0.22 | 1.39 |
| **Employment status** |  |  |
| Employed | 0.76 | 0.70 |
| Not Employed | 0.84 | 0.72 |
| **Marital Status** |  |  |
| Never married | 0.95 | 1.20 |
| Currently married | 0.80 | 0.72 |
| Formerly married | 0.94 | 0.73 |
| **Region** |  |  |
| North | 0.87 | 0.95 |
| Central | 0.83 | 0.82 |
| East | 1.07 | 1.03 |
| West | 0.69 | 0.69 |
| South | 0.93 | 1.09 |
| North East | 0.66 | 0.71 |
| **Note(s):** *SC/ST=Scheduled castes and Scheduled Tribes; OBC=Other Backward Castes; “Formerly married” includes women who are widowed, divorced, or separated at the time of the survey; P/R ratio is the ratio between the percentage of Hypertensive women falling in poorest and the richest categories.* | | |
| **Source(s):** *Author's calculation based on NFHS-4 and NFHS-5 data.* | | |
