# Supplementary material for: Trends and socioeconomic-spatial inequalities in hypertension among Muslim women in India, 2015–2021: evidence from the National Family Health Surveys
Source: Front Public Health. 2026 Jun 11;14:1828079. doi: 10.3389/fpubh.2026.1828079 (PMC13294860; doi:10.3389/fpubh.2026.1828079)
Supplement: SUPPLEMENTARY FILE S3 — Adjusted odds-ratio forest plots for hypertension among Muslim women in India, NFHS-4 (2015–16) and NFHS-5 (2019–21). [file Supplementary_file_3.docx]

**S3: Odds ratios for hypertension among Muslim women in India across socioeconomic factors: NFHS-4 (2015-16) and NFHS-5 (2019-21).**

**
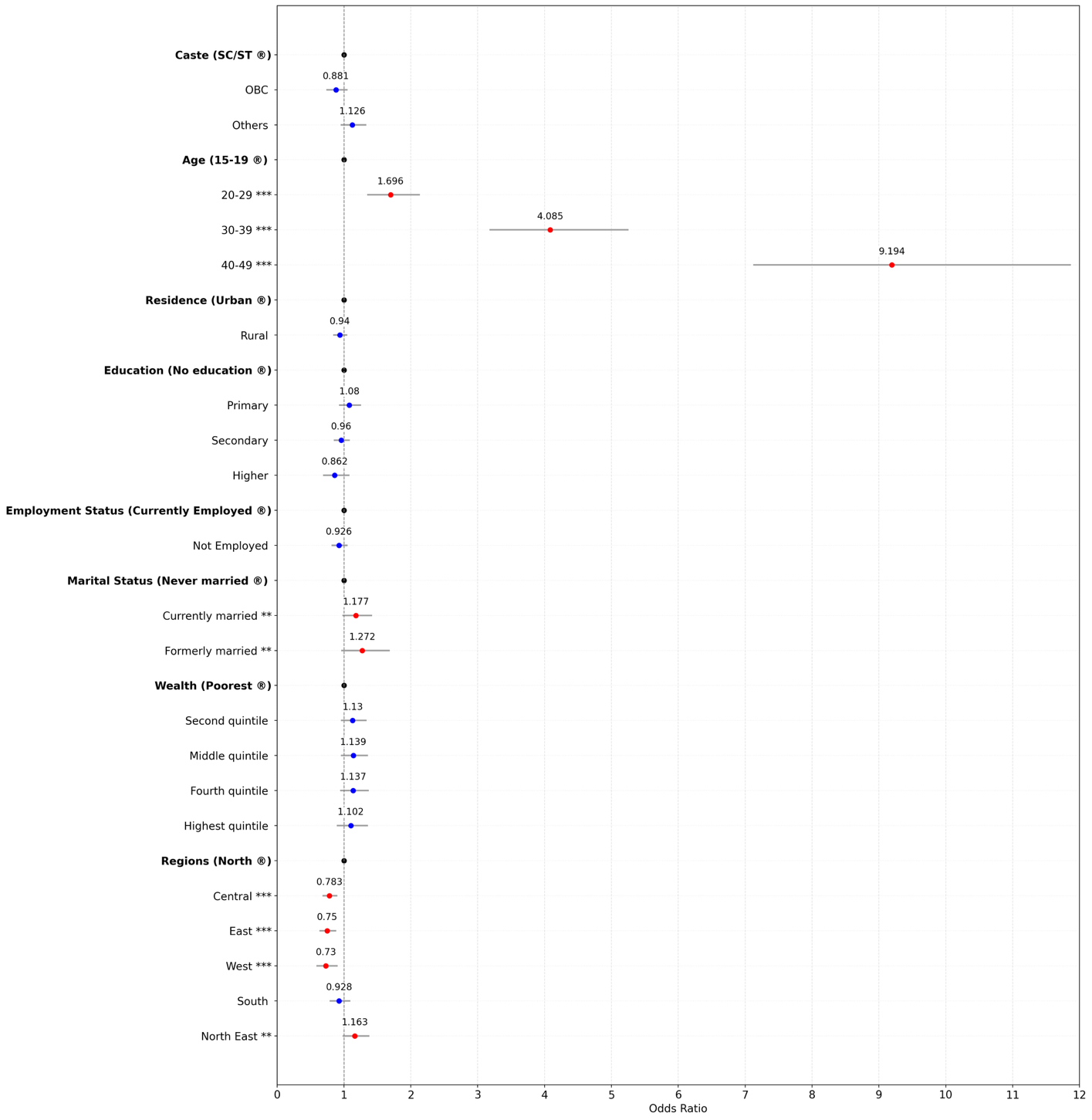
**

**NFHS-4 (2015-16)**


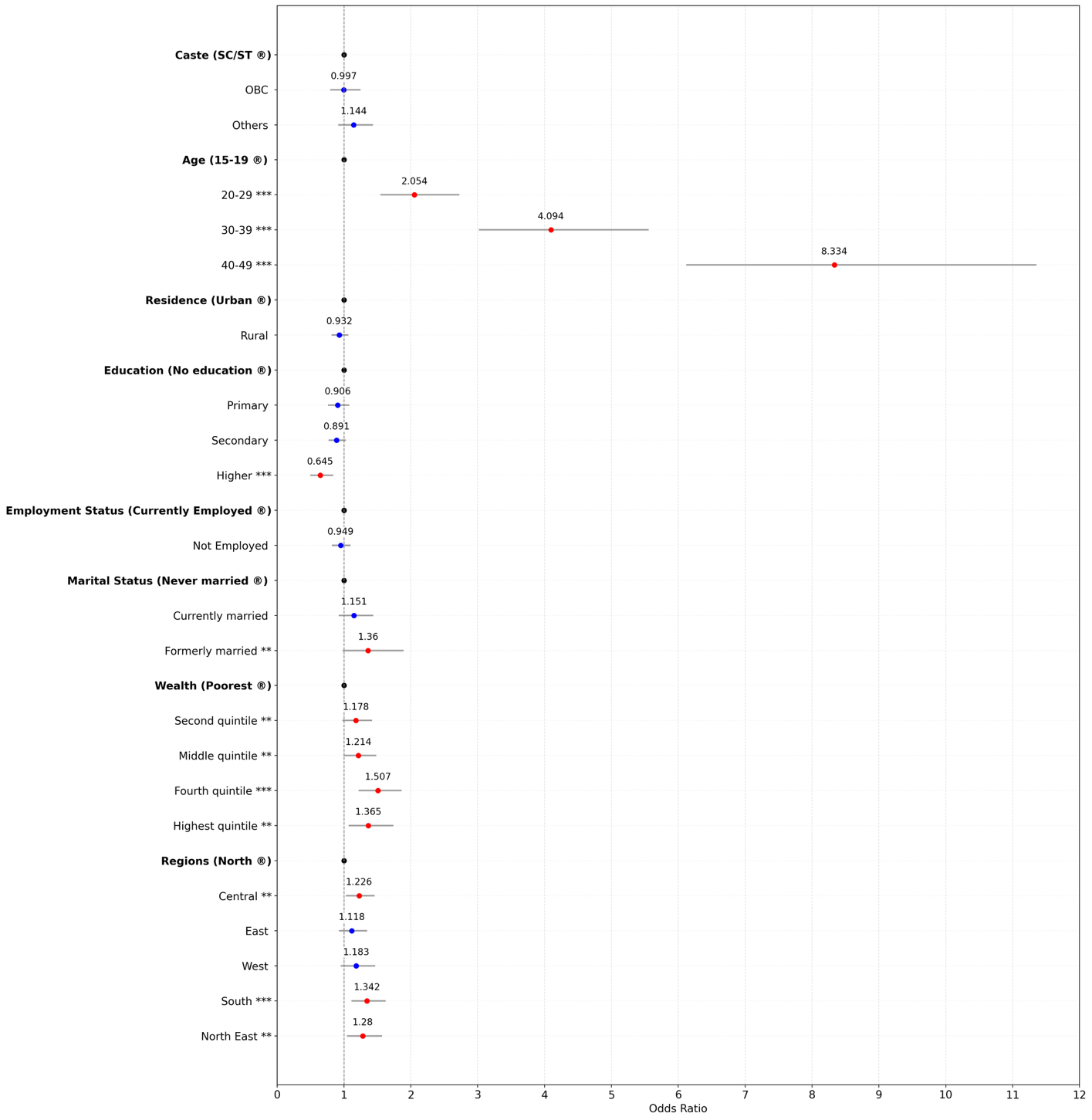


**NFHS-5 (2019-21)**

**Source:** *Author's calculation based on NFHS-4 and NFHS-5 data.*

**Notes:** SC/ST = Scheduled Castes/Scheduled Tribes; OBC = Other Backward Classes; SE = standard error; 95% conf. interval= 95% confidence interval; Estimates are survey-weighted (v005) with design adjustment for clustering (PSU v021) and stratification (v022) using svy. Reference categories (®): caste = SC/ST; age = 15–19; residence = urban; education = no education; wealth = poorest; region = North. _cons reflects the baseline odds of hypertension for the reference profile. “Formerly married” includes widowed, divorced, and separated women. Percentages/ORs may not exactly sum due to rounding. Note: p < 0.05; p < 0.01; p < 0.001. Odds ratios (OR) with 95% confidence intervals are estimated using survey-weighted multivariable logistic regression, accounting for complex survey design.
